# Progression and regression of left ventricular hypertrophy and myocardial fibrosis in a mouse model of hypertension and concomitant cardiomyopathy

**DOI:** 10.1186/s12968-020-00655-7

**Published:** 2020-08-06

**Authors:** Jacek Kwiecinski, Ross J. Lennen, Gillian A. Gray, Gary Borthwick, Lyndsey Boswell, Andrew H. Baker, David E. Newby, Marc R. Dweck, Maurits A. Jansen

**Affiliations:** 1 Centre for Cardiovascular Science, University of Edinburgh, The Chancellor’s Building, 49 Little France Crescent, Edinburgh, EH16 4SB UK; 2 Centre for Reproductive Health, University of Edinburgh, The Queen’s Medical Research Institute, 47 Little France Crescent, Edinburgh, EH16 4TJ UK

**Keywords:** Hypertension, Cardiovascular magnetic resonance, ECV, T1-mapping

## Abstract

**Background:**

Myocardial fibrosis is observed in multiple cardiac conditions including hypertension and aortic stenosis. Excessive fibrosis is associated with adverse clinical outcomes, but longitudinal human data regarding changes in left ventricular remodelling and fibrosis over time are sparse because of the slow progression, thereby making longitudinal studies challenging. The purpose of this study was to establish and characterize a mouse model to study the development and regression of left ventricular hypertrophy and myocardial fibrosis in response to increased blood pressure and to understand how these processes reverse remodel following normalisation of blood pressure.

**Methods:**

We performed a longitudinal study with serial cardiovascular magnetic resonance (CMR) imaging every 2 weeks in mice (*n* = 31) subjected to angiotensin II-induced hypertension for 6 weeks and investigated reverse remodelling following normalisation of afterload beyond 6 weeks (*n* = 9). Left ventricular (LV) volumes, mass, and function as well as myocardial fibrosis were measured using cine CMR and the extracellular volume fraction (ECV) s.

**Results:**

Increased blood pressure (65 ± 12 vs 85 ± 9 mmHg; *p* < 0.001) resulted in higher indices of LV hypertrophy (0.09 [0.08, 0.10] vs 0.12 [0.11, 0.14] g; *p* < 0.001) and myocardial fibrosis (ECV: 0.24 ± 0.03 vs 0.30 ± 0.02; *p* < 0.001) whilst LV ejection fraction fell (LVEF, 59.3 [57.6, 59.9] vs 46.9 [38.5, 49.6] %; *p* < 0.001). We found a strong correlation between ECV and histological myocardial fibrosis (r = 0.89, p < 0.001).

Following cessation of angiotensin II and normalisation of blood pressure (69 ± 5 vs baseline 65 ± 12 mmHg; *p* = 0.42), LV mass (0.11 [0.10, 0.12] vs 0.09 [0.08, 0.11] g), ECV (0.30 ± 0.02 vs 0.27 ± 0.02) and LVEF (51.1 [42.9, 52.8] vs 59.3 [57.6, 59.9] %) improved but remained impaired compared to baseline (*p* < 0.05 for all). There was a strong inverse correlation between LVEF and %ECV during both systemic hypertension (r = − 0.88, *p* < 0.001) and the increases in ECV observed in the first two weeks of increased blood pressure predicted the reduction in LVEF after 6 weeks (r = − 0.77, *p* < 0.001).

**Conclusions:**

We have established and characterized angiotensin II infusion and repeated CMR imaging as a model of LV hypertrophy and reverse remodelling in response to systemic hypertension. Changes in myocardial fibrosis and alterations in cardiac function are only partially reversible following relief of hypertension.

## Introduction

Pressure overload heart disease with concomitant myocardial fibrosis is observed in multiple common cardiac conditions including hypertension and aortic stenosis [1–8]. While there is growing evidence that excessive fibrosis is associated with adverse clinical outcomes, longitudinal human data regarding changes in left ventricular (LV) remodelling and fibrosis over time are sparse. The lack of such data is largely due to the natural history of pressure overload heart disease, with conditions such as hypertension and aortic stenosis progressing slowly over many years and decades thereby making longitudinal studies challenging. In addition, little is known about adverse remodelling reversibility once the trigger is removed [9]. Such insights are of particular interest in aortic stenosis where there is growing interest in optimizing the timing of aortic valve replacement ideally using novel objective biomarkers of LV decompensation [10–13].

Rodent models of pressure overload cardiomyopathy have been developed and potentially allow monitoring of disease progression, from the onset of pressure overload out to the advanced stages of heart failure [14–16]. These models can therefore provide longitudinal data that are lacking in humans and can potentially do so using advanced imaging techniques that can be directly translated in to humans [17, 18]. One of the most widely used models of pressure overload heart disease is transverse aortic constriction. Unfortunately, this approach involves a high dropout rate of animals related to surgery and with the band placed distal to the innominate artery causes coronary congestion and a disease state more similar to aortic coarctation than aortic stenosis or hypertension [9]. Furthermore, removal of the band is challenging, and results in incomplete relief of the obstruction making the study of reverse remodelling difficult [19]. Consequently, only a few studies have investigated reverse remodelling upon removal of the pressure overload and these did not include advanced imaging techniques [20–22].

The purpose of this study was to develop and characterize a mouse model of reversible hypertension induced cardiomyopathy and to use state-of-the-art in vivo imaging to interrogate LV hypertrophy, myocardial fibrosis, and cardiac function both before and after afterload reversal. Ultimately, our aim was to create a model that might help develop novel non-invasive biomarkers of LV decompensation in response to pressure overload and provide insight in to the time-course of clinically relevant conditions.

## Methods

### Study protocol

**Fig. 1:**
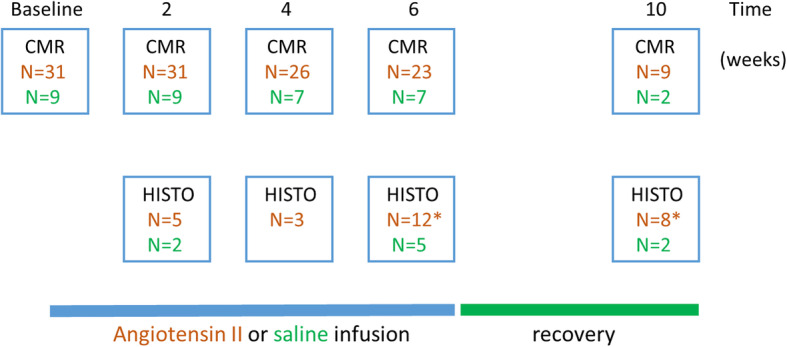
Schematic diagram of study the study protocol. MRI was performed at baseline, during Angiotensin-II infusion (2, 4 and 6 weeks) and after a 4-week recovery period. Animals were sacrificed at these time points and hearts analysed using histology (HISTO) methods. Control animals are depicted in green and Angiotensin-II treated animals in brown. * Due to a tissue fixation error 2 hearts from animals sacrificed at 6 weeks and 1 heart from the 10 week cohort was not suitable for staining and histological analysis

Thirty-one 14 ± 2 weeks, male C57BL/6 J mice (Envigo, Indianapolis, Indiana, USA) were subjected to a continuous angiotensin II infusion at 480 ± 34 ng/kg/min for up to 6 weeks (Fig. 1). A subset (*n* = 9) of the animals receiving a 6-week infusion were assessed for an additional 28 days after discontinuation of the infusion to allow investigation of reverse remodelling. Baseline measurements were taken at the start of the study, and before any intervention so that each animal could serve as its own control and intra-subject analysis could be performed. A control cohort of animals (14 ± 2 week male C57BL/6 J mice) received a placebo infusion of saline (0.9% NaCl) (*n* = 9) and was included to study and correct for any age- and procedure-related effects not attributable to angiotensin II. Throughout the experiments, animals were on a regular chow diet, housed 5 mice per cage in a 12 h:12 h light/dark cycle in animal facilities at the University of Edinburgh.

### Animal model of systemic hypertension

To subject animals to systemic hypertension, subcutaneously implanted osmotic minipumps (Alzet, Cupertino, California, USA) were used [23]. Minipumps were prepared to deliver angiotensin II (Ang-II, Sigma-Aldrich, Darmstadt, Germany) at predetermined rates of infusion [22]. Implantation was performed surgically under inhalational anaesthesia (isoflurane (1.5–2%), supplemental data). To monitor pressure overload, animals underwent serial blood pressure measurements using a Letica LE 5002 Non-Invasive Blood Pressure System (Panlab, Cornellá, Spain) which operates on the same basis as clinically used sphygmomanometers (tail cuff method). Prior to study commencement, mice were trained for tail cuff plethysmography in order to decrease animal stress related to measurement acquisition and to improve reproducibility. During the experimental period, mice were subjected to plethysmography after each imaging session. After 2 preliminary measurements, at least three recordings of systolic, diastolic and mean arterial pressure were recorded in each animal.

### Cardiovascular magnetic resonance imaging

All animals underwent serial cardiovascular magnetic resonance (CMR) imaging to assess LV structure and function as well as myocardial fibrosis. CMR was performed initially at baseline, before the onset of hypertension (so that progression of adverse remodelling could be compared to baseline), and then every 2 weeks during angiotensin II infusion. In the reverse remodelling subgroup, a final CMR scan was performed 4 weeks after the angiotensin II infusion had been discontinued. At the end of the final imaging session, animals were sacrificed by exsanguination under deep anaesthesia with the heart stored for further histological analysis.

All CMR scans were performed on a 7 T preclinical horizontal-bore CMR system (Agilent Technologies, Santa Clara, CA, USA) equipped with a 33-mm inner diameter quadrature radiofrequency coil for mice (Rapid Biomedical, Rimpar, Germany). Animals were anaesthetised using isoflurane (3% isoflurane in 1.0 L/min oxygen for induction and 1.5–2% isoflurane in 0.5 L/min O_2_/0.5 L/min air for maintenance). Prior to imaging, an Anicath 26G cannula (Millpledge Veterinary, Clarborough, UK) was placed intraperitoneally for administration of the gadolinium-based contrast agent (gadobenate dimeglumine 1 μL/g body weight [Multihance]; Bracco Diagnostics, Princeton, New Jersey, USA). Animals were placed in a CMR cradle with electrocardiogram (ECG) leads placed subcutaneously. The animals were positioned supine with hearts in the centre of the coil, and in the centre of the CMR scanner. A pressure transducer was placed under the torso for constant monitoring of respiration (CMR-compatible Small Animal Monitoring and Gating System, SA Instruments, Stony Brook, New York, USA), and a rectal probe was inserted to monitor and maintain body temperature at 37 ± 0.5 °C via a feedback-controlled warm air system (CMR-compatible Small Animal Heating System, SA Instruments). Throughout the imaging session we adjusted the warm air system to maintain target body temperature and as a result a stable heart rate at 530–600 beats/min and a respiratory rate of 45–75 breaths/min.

Scout images were taken to confirm correct positioning and to orientate 1.0-mm short axis slices covering the heart. T1 mapping for calculation of regional myocardial T1 relaxation times was accomplished using a gradient-echo, cardiac-gated modified Look-Locker inversion recovery sequence (MoLLI) during which 30 images of a single mid-ventricular slice were acquired at unique inversion times (dependent on heart rate, ranging from approximately 0.10 to 3.00 s) with the recovery time after each slice-selective inversion pulse of about 4.5 s, as described previously [24, 25]. Imaging readout was with a cardiac fast gradient echo (repetition time 2.8 ms; echo time 1.4 ms; flip angle 10°, matrix 128 × 128; field of view 30 × 30 mm^2^; in-plane resolution = 0.23 × 0.23 mm^2^; trigger delay 0.5 × R-R; slice thickness 1.5 mm;). We acquired eight phase-encoding echos per segment and 8 signal averages were used to compensate for respiratory motion. After acquisition of a baseline T1 map, gadobenate dimeglimine was injected via the intraperitoneal cannula, and a second T1-mapping dataset was acquired 20 min post-injection. During the time between the T1-mapping acquisitions, cine images were acquired for structural and functional assessment. The short axis, vertical and horizontal long axis cardiac images were acquired using an ECG triggered and respiratory-gated gradient echo sequence (TR/TE = 5.2/1.3 ms, flip angle: 15°) with gradient and radiofrequency spoiling. Eighteen phases were acquired with a field of view of 30 × 30 mm, a 128 × 128 matrix, and 2 signal averages were used. For the LV short axis, nine consecutive 1-mm-thick slices were acquired, which encompassed the entire heart from base to apex. Late gadolinium enhancement (LGE) imaging was performed between 12 and 18 min after contrast administration with a short-axis cardiac-triggered/respiratory-gated T1-weighted gradient echo inversion-recovery scan with the following imaging parameters: TR: 1000 ms (depending on respiration rate), TE: 1.45 ms, field of view: 30 × 30 mm, number of signal averages: 4, inversion time: 560 ms, flip angle: 90°, nine 1-mm thick slices covering the entire left ventricle.

### Image analysis

Data were analysed using cvi42 software (Circle CVI, Calgary, Alberta, Canada). The short axis cine LV images were used for the assessment of LV volumes, mass and ejection fraction. The epicardial and endocardial contours were carefully identified and planimetered in end-systole and end-diastole for LV volume quantification. The LV mass was calculated from the total myocardial volume (excluding trabeculations and papillary muscles) multiplied by the density of the myocardium (1.05 g/mL). Maximal LV wall thickness was evaluated in all 17 LV segments from cine images of the LV in end-diastole [26]. For LV diffuse fibrosis, we calculated the extracellular volume fraction (ECV) derived from pre- and post-contrast myocardial T1 values corrected for blood-pool T1. The ECV% was calculated according to: ECV = partition coefficient × [1-hematocrit]; where partition coefficient = [∆R1myocardium/∆R1blood-pool] and ∆R1 = (1/post-contrast T1–1/pre-contrast T1). To avoid blood-pool contamination and partial volume effects, we used a mid-myocardial region of interest for the T1-mapping analysis. A two-pixel wide myocardium and ventricular boundary zone was excluded from all applicable slices. We actively searched for areas of LGE within the myocardium.

### Histology

Histological validation was performed for each CMR technique used to measure myocardial fibrosis. After the final imaging session, animals were euthanized by exsanguination under isoflurane anaesthesia. The heart was removed and transferred into 10% neutral buffered formalin (Cellstor, CellPath, Newton, UK) for fixation for at least 20 h. After fixation, hearts were processed and embedded in paraffin in a short axis orientation so that the cut sections would all show the ventricle lumen. Slides were stained in a 0.1% sirius red solution in saturated picric acid (Picrosirius red, Sigma, Dorset, UK). All picrosirius red stained slides images were acquired on the AxioScan Z1 (Carl Zeiss, Oberkochen, Germany) and analysed using Image-Pro Premiere 9.1 (MediaCybernetics, Rockville, Maryland, USA) using a tissue specific threshold. One midventricular slice was analysed for each heart to enable correlation with T1 mapping data. The area of fibrosis was expressed as a percentage of the total myocardial area in that section (see also Supplemental Data).

### Statistical analysis

Continuous variables were tested for distribution with the Shapiro-Wilk test. Data are presented as mean ± SD, median [interquartile range] or percentages where appropriate. Differences between groups were assessed with the use of a 2-sided Student *t*-test, paired Student *t*-test, ANOVA for continuous variables, Wilcoxon rank-sum test for ordinal variables and the χ^2^ test or Fisher’s exact test for categorical data. The relationship between 2 continuous variables was assessed using the Pearson’s correlation coefficient. A 2-sided *p* < 0.05 was considered statistically significant.

## Results

### LV remodelling under systemic hypertension

Mean arterial pressure (MAP) was elevated after 2 weeks of angiotensin II infusion compared to baseline (84 ± 14 vs 65 ± 12 mmHg, *p* < 0.001) and remained stable for the remainder of the infusion phase (*p* > 0.50; Table 1). Both LV maximum wall thickness and LV mass increased during angiotensin II infusion (0.73 [0.71,0.76] vs 0.84 [0.81, 0.86] mm, *p* = 0.001 and 0.09 [0.08, 0.10] vs 0.10 [0.10, 0.12] g, *p* < 0.001 respectively). Wall thickness and LV mass were elevated after 2 weeks and increased further up to the 4-week time point (0.84 [0.81, 0.86] vs 0.88 [0.80, 0.95] mm, p < 0.001 and 0.11 [0.10, 0.12] vs 0.12 [0.11, 0.14] g, p < 0.001) after which both plateaued (Table 1). Such LV remodelling was not observed in control animals (Table 1).

**Table 1:** Left ventricular remodelling under pressure overload and after load normalization

	Baseline		2 weeks		4 weeks		6 weeks		4 weeks post-infusion	Statistics I *P* value for a trend (ANOVA)	Statistics II P value 6 week vs 4 weeks post infusion
Animals	Experimental n = 31	Control n = 9	Experimental n = 31	Control n = 9	Experimental *n* = 26	Control *n* = 7	Experimental *n* = 23	Control n = 7	Experimental n = 9		
MAP, mm Hg	65.1 ± 12.0	65.3 ± 10.8	84.2 ± 13.6	64.8 ± 11.6	86.5 ± 7.1	66.9 ± 12.1	84.7 ± 9.2	64.0 ± 13.2	68.8 ± 5.4	< 0.001	< 0.001
EDV, ml	0.058 [0.053, 0.063]	0.059 [0.054, 0.063]	0.071 [0.066, 0.081]	0.060 [0.056, 0.064]	0.074 [0.065, 0.086]	0.062 [0.060, 0.065]	0.083 [0.073, 0.097]	0.065 [0.060, 0.067]	0.081 [0.067, 0.095]	< 0.001	0.60
ESV, ml	0.022 [0.020, 0.025]	0.022 [0.020, 0.025]	0.033 [0.30, 0.040]	0.023 [0.020, 0.024]	0.041 [0.034, 0.045]	0.023 [0.020, 0.026]	0.048 [0.037, 0.055]	0.025 [0.021, 0.027]	0.041 [0.032, 0.063]	< 0.001	< 0.20
Wall Thickness, mm	0.73 [0.71, 0.76]	0.72 [0.71,0.77]	0.84 [0.81, 0.86]	0.74 [0.70, 0.79]	0.88 [0.80, 0.95]	0.73 [0.70, 0.77]	0.86 [0.82, 0.91]	0.75 [0.72, 0.80]	0.82 [0.80, 0.92]	0.001	0.18
LV mass, g	0.091 [0.083, 0.105]	0.090 [0.084, 0.100]	0.108 [0.101, 0.121]	0.089 [0.085, 0.099]	0.121 [0.111, 0.136]	0.095 [0.088, 0.100]	0.123 [0.111, 0.138]	0.099 [0.090 0.103]	0.108 [0.098, 0.116]	< 0.001	0.003
LV ECV, %	0.24 ± 0.03	0.24 ± 0.03	0.30 ± 0.04	0.24 ± 0.04	0.309 ± 0.03	0.24 ± 0.04	0.30 ± 0.02	0.25 ± 0.04	0.27 ± 0.02	0.001	0.001
Heart Rate	561 ± 30	555 ± 28	557 ± 29	553 ± 32	542 ± 37	552 ± 39	543 ± 40	553 ± 41	556 ± 38	0.74	0.58
SV, ml	0.031 [0.027, 0.035]	0.031 [0.027, 0.036]	0.033 [0.029, 0.035]	0.032 [0.029, 0.033]	0.034 [0.028, 0.036]	0.032 [0.028, 0.035]	0.033 [0.031, 0.034]	0.033 [0.031, 0.034]	0.035 [0.031, 0.036]	0.19	0.70
LVEF, %	59.3 [57.6, 59.9]	59.0 [56.5, 60.8]	52.4 [47.7, 53.9]	58.7 [57.1, 60.9]	49.5 [41.2, 51.7]	59.5 [56.2, 61.7]	46.9 [38.5, 49.6]	60.2 [56.9, 61.5]	51.1 [42.9, 52.8]	< 0.001	0.10

ECV – extracellular volume , EDV – end diastolic volume, ESV – end diastolic volume, LVEF- left ventricular ejection fraction, SV – stroke volume. Statistics I: Repeated Measures ANOVA (baseline, 2 weeks, 4 weeks, 6 weeks). Statistics II: Paired student T test (6 weeks versus 1 month post infusion)

### Histological assessment of myocardial fibrosis

**Fig. 2:**
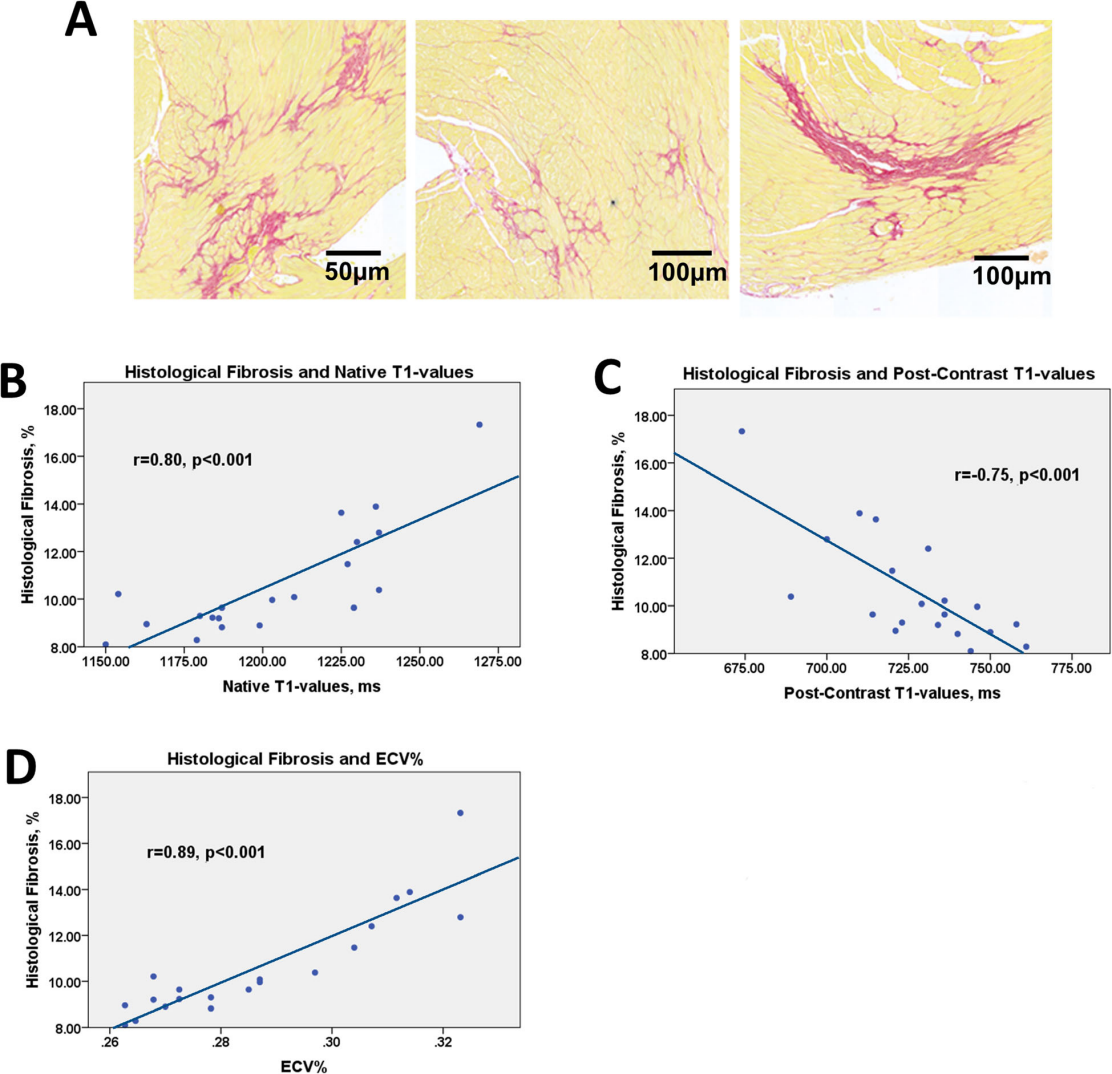
Fibrosis on histology and T1-mapping data. Examples of diffuse fibrosis in mice subjected to systemic hypertension for 6 weeks on histology - picrosirius red staining (A). The relationship between fibrosis on histology and the native T1-values (B), post contrast T1-values (C) and the extracellular volume (D). While native T1 and ECV showed a positive correlation with histological fibrosis due to kinetics of the contrast agent’s myocardial clearance, post-contrast T1 values are negatively correlated with histological fibrosis. Hearts from 20 hypertensive animals sacrificed immediately after imaging (during the infusion of angiotensin II at the 2-week (*n* = 5), 4-week (*n* = 3) and 6-week (*n* = 12) timepoint) were used for this analysis. Pearson’s correlation coefficient

Hearts from 28 animals were available for histological analysis and validation of the CMR fibrosis measurements (Fig. 1, Fig. 2A). While control animals showed no difference in fibrosis over time we observed an increase in myocardial fibrosis in animals exposed to the angiotensin II infusions compared to control animals (4.4, [3.5, 5.8] vs 10.1 [8.7, 12.9] % and baseline (4.5, [3.5, 5.8] vs 10.1 [8.7, 12.9], both *p* < 0.001). There was a strong correlation between the histological assessment of myocardial fibrosis (% myocardium comprised of fibrosis) and the ECV% as measured by CMR (r = 0.89, *p* = 0.001; Fig. 2). Similar correlations were observed between histological fibrosis and native T1 (r = 0.80, p < 0.001) and post contrast T1 (r = − 0.75, p < 0.001).

### LV fibrosis and function under systemic hypertension

**Fig. 3:**
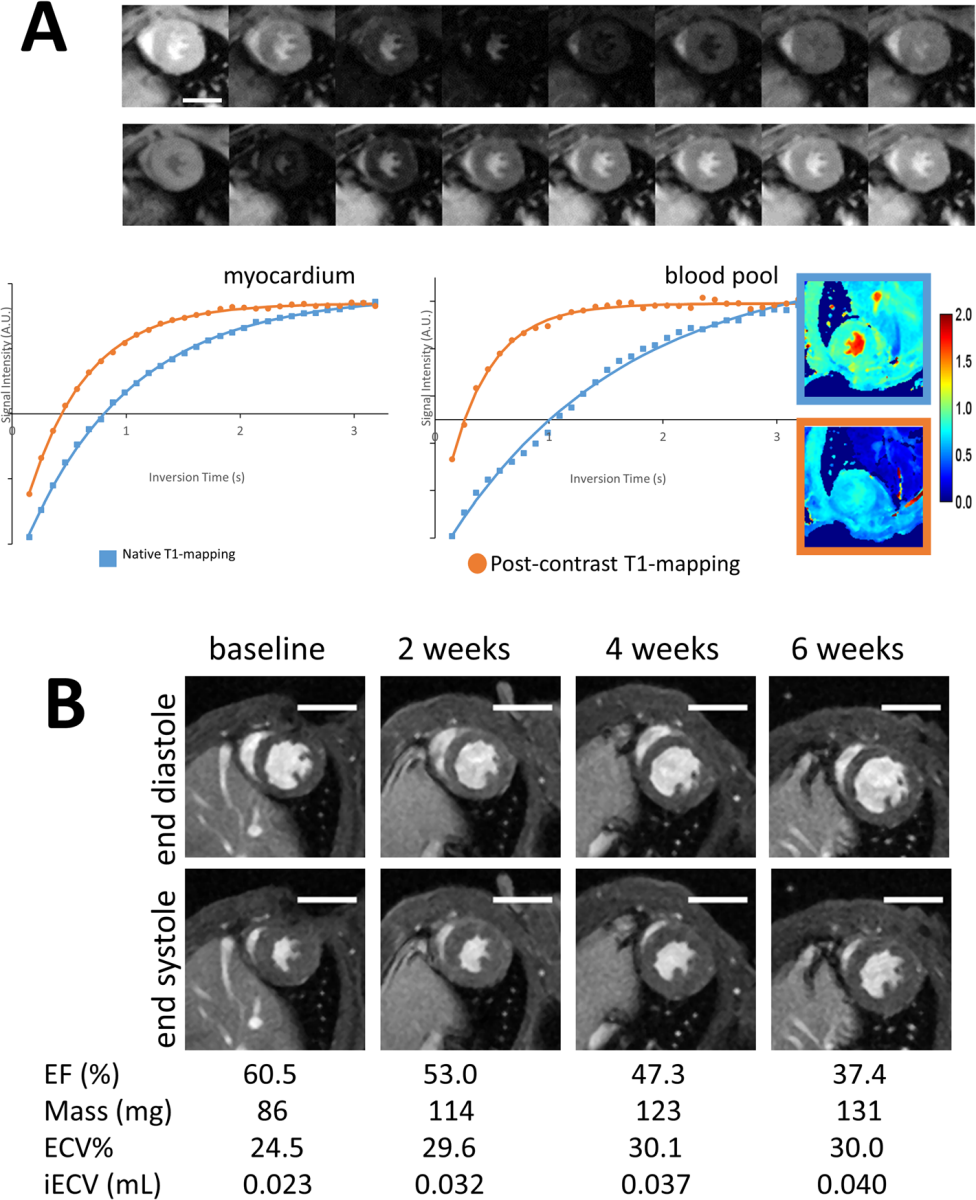
Cardiovascular magnetic resonance imaging of mice subjected to systemic hypertension. Modified Look-Locker Inversion Recovery T1-mapping (A). T1 relaxation times for the myocardium and bloodpool are generated from images shown in the upper row. The calculated T1 relaxation times can be used to generate native (**blue**) and postcontrast (**orange**) T1 maps. End-diastolic and end-systolic LV short axis images demonstrating adverse remodelling due to an increased afterload (B). The actual length of the scale bar is 5 mm

**Fig. 4:**
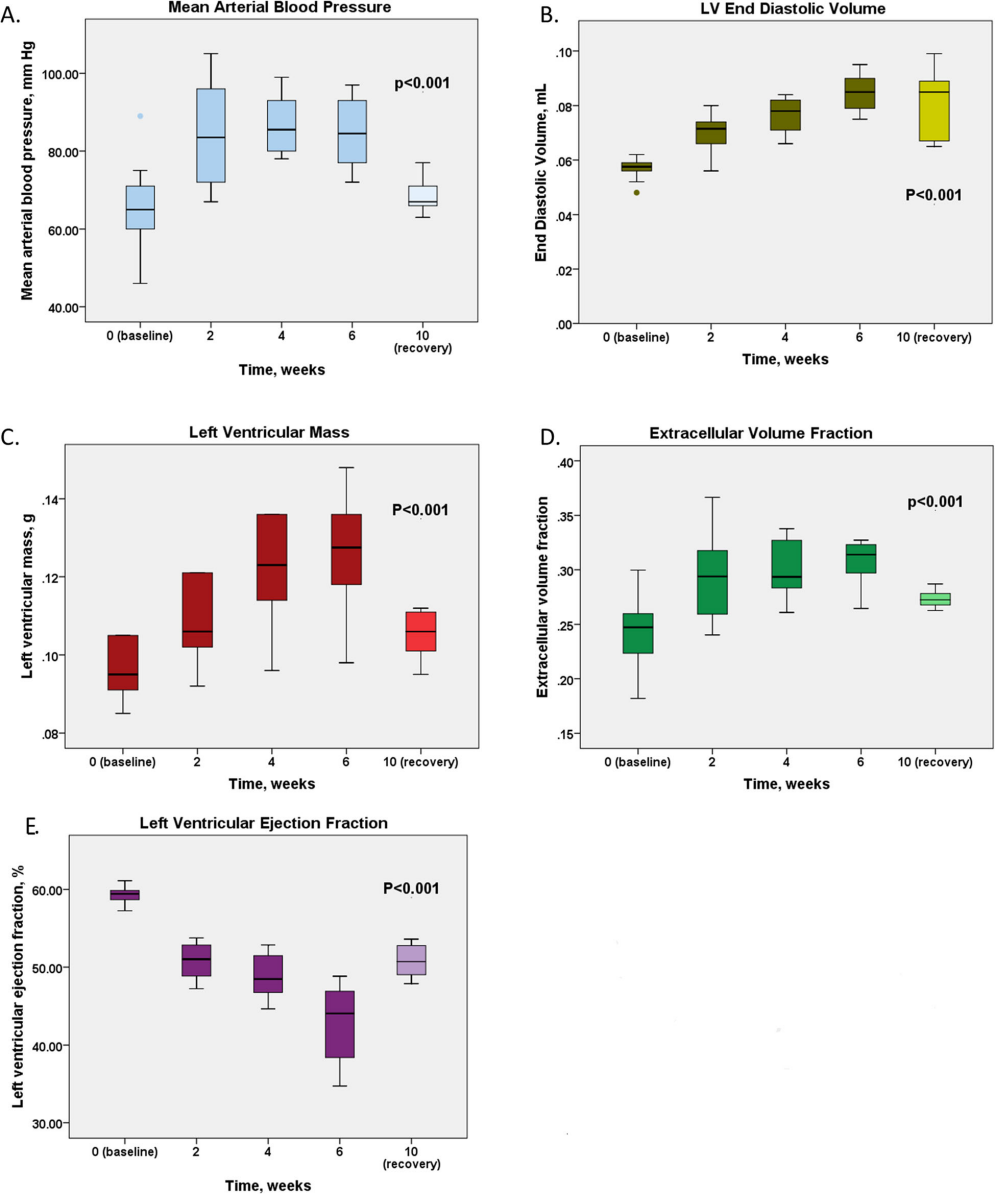
Adverse remodelling under systemic hypertension. Mean arterial pressure (A), Left ventricular end diastolic volume (B), Left ventricular mass (C), Extracellular volume fraction (D), LV ejection fraction (E). Thirty-one experimental animals underwent imaging at baseline and 9 animals were followed in the recovery phase, the exact number of mice scanned at each timepoint is presented in Fig. 1. Repeated measures ANOVA (baseline, 2 week, 4 weeks, 6 weeks). For post-hoc comparisons, see Table 1

While the changes in native T1 relaxation times during exposure to systemic hypertension did not reach statistical significance (*p* = 0.10), ECV demonstrated progressive increases. This increase in ECV demonstrated a similar pattern of change as the blood pressure with an initial increase (0.24 ± 0.03 vs 0.30 ± 0.04, *p* = 0.004) followed by a plateau phase after 4 weeks (*p* > 0.40). Consistent with this, LVEF also demonstrated an ongoing gradual reduction with time spent on the angiotensin II infusion (LVEF 59.3 [57.6, 59.9] % at baseline, 52.4 [47.7, 53.9] % at 2 weeks, 49.5 [41.2, 51.7] % at 4 weeks and 46.9 [38.5, 49.6] % at 6 weeks; *p* < 0.001 for trend) (Figs. 3 and 4). Within our study population we did not observe any myocardial late gadolinium enhancement indicative of focal replacement fibrosis.

### Reverse remodelling

Four weeks after the end of the angiotensin II infusion, reversal of the pressure overload state was observed with mean arterial blood pressure values returning to baseline (*p* = 0.42 for difference, Fig. 4A). In response to the normalisation of blood pressure, LV mass and ECV all regressed (*p* < 0.01; Table 1). However, this reverse remodelling was only partial with none of these measures returning to their baseline values (Fig. 4). Indeed, ECV only partially resolved, with values reducing from 0.30 ± 0.02 during the infusion to 0.27 ± 0.02 (*p* = 0.001) 4 weeks following its discontinuation compared to baseline values of 0.24 ± 0.03 (*p* = 0.06). Importantly, the LVEF again demonstrated a similar pattern to ECV, showing a trend towards recovery (46.9 [38.5, 49.6] vs 51.1 [42.9, 52.8] %, *p* = 0.10) yet remaining lower at 4 weeks than at baseline (59.3 [57.6, 59.9] vs 51.1 [42.9, 52.8] %, *p* = 0.029; Table 1). The LV EDV remained elevated: 0.08 [0.07, 0.10] mL compared to 0.08 [0.07, 0.10] mL during pressure overload (*p* = 0.70).

### ECV% is associated with systolic function

There was a moderate inverse correlation between LVEF and ECV% values during pressure loading (r = − 0.54, *p* < 0.001 at 6 weeks, Table 2). On multivariable linear regression analysis, ECV% was independently associated with LVEF independent of MAP and LV mass (Table 2).

**Table 2:** Univariable and multivariable linear regression analysis to examine association of variables with LV ejection fraction after 6 weeks of pressure overload

Variables	Univariable		Multivariable – Model 1 (included early ECV% change) R-Sq = 0.62		Multivariable – Model 2 (included ECV% at 6 weeks) R-Sq =0.54	
	Relative change in LVEF (95% CI)	P- value	Relative change in LVEF (95% CI)	P- value	Relative change in LVEF (95% CI)	P- value
MAP, per 5 mmHg	−2.8 (−5.4- -0.1)	0.04	−1.3 (3.0–0.4)	0.23	−1.9 (−3.9–0.1)	0.11
LV mass, per 1 mg	0.1 (−0.05–0.3)	0.08	−0.2 (−0.5–0.1)	0.27	0.2 (−0.1–0.5)	0.21
Early change in ECV%, per 1%	− 2.1 (− 2.9- -1.3)	< 0.001	−2.2 (− 2.9- -1.5)	< 0.001		
ECV%	− 1.6 (− 2.3- -0.8)	< 0.001			−1.5 (− 2.2 - -0.7)	< 0.001

ECV% – extracellular volume fraction, LVEF – left ventricular ejection fraction, MAP – mean arterial pressure

**Fig. 5:**
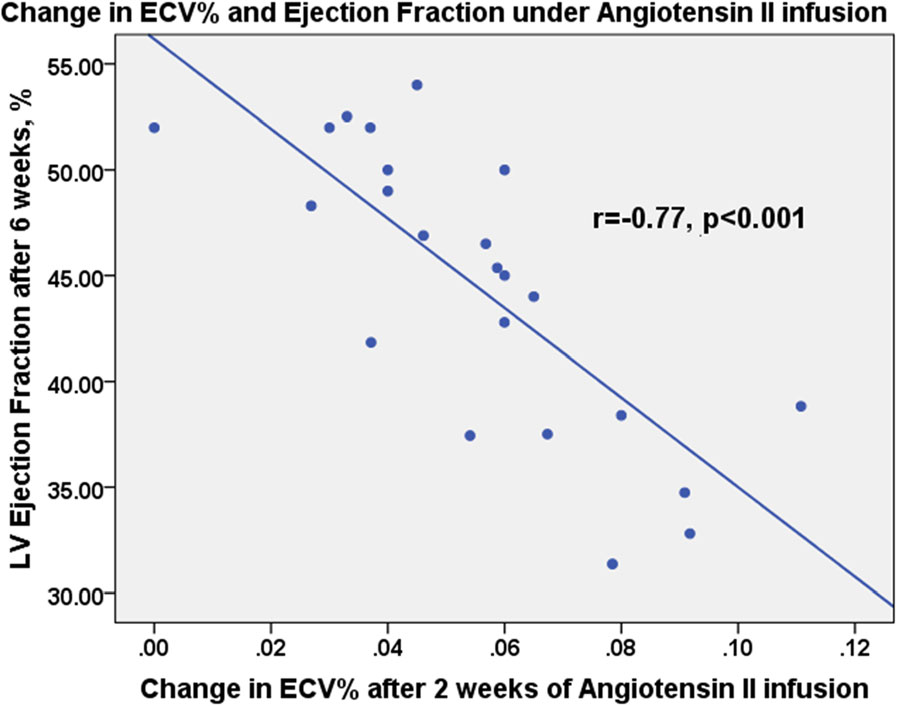
Diffuse fibrosis and systolic function in 23 animals which were subjected to a continuous angiotensin II infusion for 6 weeks. Early changes in ECV predict later reductions in LVEF. Pearson’s correlation coefficients

We found a strong correlation between the increases in ECV observed in the first two weeks of systemic hypertension (from baseline to week 2) and the reduced LVEF after 6 weeks (r = − 0.77, p < 0.001; Fig. 5).

## Discussion

In this study, we have used the angiotensin II infusion model of hypertension to establish an animal model of reverse remodelling following load normalization. This model has allowed us to assess how the hypertrophic response and myocardial fibrosis develop in response to an increased afterload and then reverse remodels once that stimulus is removed. We believe our model will allow improved understanding of the processes underlying LV decompensation in patients with aortic stenosis and hypertension. In this study, we have demonstrated that myocardial fibrosis appears to be the primary determinant of LV dysfunction both before and after reversal of systemic hypertension. Further studies should investigate whether reversing systemic hypertension earlier might potentially lead to complete recovery in LV function and whether the optimal timing can be identified using imaging.

Our preclinical model offers several important advantages. Using state-of-the-art in vivo CMR imaging, we have been able to obtain longitudinal data on myocardial remodelling within a timespan of months rather than years or decades as observed in humans. Furthermore, it facilitates histological correlation with imaging findings, enabling validation of our CMR markers of fibrosis, including ECV, as biomarkers of LV fibrosis. Finally, our preclinical model provides insights into both the hypertrophic response and reverse remodelling using state-of-the-art longitudinal imaging assessments. While several studies have utilized transverse aortic constriction as a model of aortic stenosis, this model has several limitations [14–16]. The surgical technique is associated with a 25% drop-out of animals in the peri-procedural period [9]. With the aortic band placed between the origin of the innominate and left common carotid arteries the model recreates proximal aortic coarctation leading to significant hemodynamic consequences. These include elevated pressure in the innominate, right common carotid, coronary and right subclavian arteries as opposed to the left subclavian and carotids. In severe constriction, perfusion of organs distal to the band can be maintained through the Willis circle yet this again is not typical for aortic valve stenosis or hypertension.

Continuous infusion of angiotensin II leads to systemic hypertension and holds major promise in improving our understanding of LV decompensation in pressure overload conditions and in the development of novel biomarkers and treatment strategies [27]. Indeed, in this study, we have demonstrated that reductions in ejection fraction associated with hypertension are more closely associated with the total burden of myocardial fibrosis than the degree of pressure afterload. Moreover, we observed that reverse remodelling 1 month after load normalization is incomplete with residual fibrosis again closely associated with residual systolic impairment. Our findings are in line with the hypothesis that fibrosis is the principal driver of LV decompensation in pressure overload [7], a key determinant of LV systolic function and an important potential therapeutic target. This is a particularly appealing target given that several drug therapies have already demonstrated their ability to reduce myocardial fibrosis in other contexts [1, 28]. Indeed, our model could potentially be used to assess the efficacy of existing or novel anti-fibrotic therapies before proceeding to expensive clinical trials.

This model might prove of value in determining the optimal timing of aortic valve replacement. The data presented here suggest that if pressure overload (hypertension) continues for too long then irreversible fibrosis will develop in the LV, leading to long term impairment of systolic function. Further studies should investigate whether reversing pressure overload earlier might potentially lead to complete recovery in LV function and whether the optimal timing can be identified using imaging or other biomarkers. This strategy could ultimately then be applied to patients with aortic stenosis, thereby optimizing the timing of aortic valve replacement.

### Limitations

We acknowledge that our study has limitations. While our model recreates the myocardial response to increased afterload observed in patients with hypertension and aortic stenosis, it is not a pure model for either. Moreover, angiotensin II might have triggered diffuse fibrosis irrespective of the elevated blood pressure [29]. For T1-mapping, we only acquired data from the mid-ventricle short axis slice potentially introducing a sampling error. In patients, data are usually obtained from 3 slices so that the basal, mid and apical segments are all interrogated. Since our model of hypertension affects the entire LV and due to animals’ welfare, we chose to shorten the image acquisition by focusing on the mid-ventricle slice. We performed blood pressure measurements immediately after the imaging sessions at the time animals were recovering from anaesthesia. We found such an approach to be less stressful for mice whilst allowing for recording multiple readings. Finally, we only assessed reverse remodelling at 4 weeks post cessation of the angiotensin II infusion, therefore we cannot rule out that the fibrosis burden and LV mass would normalise during longer follow-up.

## Conclusions

Using state-of-the-art in vivo CMR imaging, we have developed a mouse model of LV hypertrophy and reverse albeit incomplete remodelling in response to systemic hypertension which allows tracking of changes in LV remodelling, myocardial fibrosis and systolic function. In this study, we have demonstrated that myocardial fibrosis appears to be the primary determinant of LV dysfunction both before and after reversal of pressure overload.

## Supplementary information

**Additional file 1.**

## Data Availability

The imaging protocols as well as the datasets used and/or analysed during the current study are available from the corresponding author on reasonable request.

## Abbreviations

CMR: Cardiovascular magnetic resonance
ECV: Extracellular volume
EDV: End-diastolic volume
ESV: End-systolic volume
LGE: Late gadolinium enhancement
LV: Left ventricle/left ventricular
LVEF: Ejection fraction
MAP: Mean arterial pressure
MOLLI: Modified Look-Locker inversion recovery
SV: Stroke volume
TD: Delay time
TE: Echo time
TR: Repetition time
