# Supplementary material for: Progression and regression of left ventricular hypertrophy and myocardial fibrosis in a mouse model of hypertension and concomitant cardiomyopathy
Source: J Cardiovasc Magn Reson. 2020 Aug 6;22:57. doi: 10.1186/s12968-020-00655-7 (PMC7409657; doi:10.1186/s12968-020-00655-7)
Supplement: Supplementary file 1 — Additional file 1. [file 12968_2020_655_MOESM1_ESM.docx]

**Supplemental Data**

Progression and regression of left ventricular hypertrophy and myocardial fibrosis in a mouse model of hypertension and concomitant cardiomyopathy

Jacek Kwiecinski^1^, Ross J. Lennen^1^, Gillian A. Gray^1^, Gary Borthwick^1^, Lyndsey Boswell^2^, Andrew Baker^1^, David E. Newby^1^, Marc R. Dweck^1^, Maurits A. Jansen^1^

^1^Centre for Cardiovascular Science, University of Edinburgh, United Kingdom

^2^Centre for Reproductive Health, University of Edinburgh, United Kingdom

Corresponding author:

Maurits Jansen

Centre for Cardiovascular Science

The Chancellor's Building

49 Little France Crescent

Edinburgh EH16 4SB

[m.a.jansen@ed.ac.uk](mailto:m.a.jansen@ed.ac.uk)

Contents:

1. Methods

- Osmotic Minipump Preparation
- Surgical Procedures
- Blood Pressure Measurements
- Sacrifice and Tissue Collection
- Histological Analysis

1. Figure S1. Schematic diagram of study the study protocol

Osmotic Minipump Preparation

To subject animals to pressure overload subcutaneously implanted osmotic minipumps (Alzet, Cupertino, CA) were used. These minipumps operate on the basis of an osmotic pressure difference between the pump’s osmotic compartment and the surrounding tissue environment. They consist of a fluid reservoir (containing the drug) which is encapsulated by an osmotic layer covered by a semipermeable membrane. The high osmolality of the osmotic layer attracts water from the surrounding environment and this influx leads to compression of the reservoir chamber and guarantees a continuous delivery of the solution at a predefined rate which is specific for each pump model {theeuwes 1976, Ann Biomed Eng, 4, 343-53}. Minipumps were prepared to deliver Angiotensin II (Sigma-Aldrich, Darmstadt, Germany) at the rate of 480±34 ng/kg body weight/min. The total dose of Angiotensin II was calculated considering the pump reservoir size, flow rate and the animal weight. All pumps were filled using the provided 25-gauge filling needle (Alzet, Cupertino, CA) and a 1mL syringe. Special care was taken to avoid transferring any air bubbles into the filling reservoir as these might impede drug delivery. To confirm that no substantial air bubbles were present in the solution once filled all pumps were weighed. Control animals (n=9) were implanted with osmotic minipumps filled with sterile saline.

Surgical Procedures

Subcutaneous minipump implantation was performed under a sterile technique. To anaesthetise the animal, the induction chamber was filled with 3% isoflurane in oxygen (flow rate: 1L/min) and the mouse was placed in the box. When induction was confirmed (by unresponsiveness to external stimuli and absence of the palpebral reflex) the animal was placed on a heated mat (to maintain body temperature at 37°C) under a face mask delivering 2-2.5% isoflurane in oxygen (flow rate 1L/min). Hair was removed from the interscapular part of the animals’ back using a hair trimmer. Afterwards any residual hair was removed diligently with the vetasept chlorhexidine surgical disinfectant. A small horizontal incision was made in the interscapular region using sharp surgical scissors. Subsequently using blunt, straight dissection forceps a pocket under the skin was created for the placement of the Alzet minipump. In the next step the minipump was inserted into the newly established subcutaneous pocket with the flow moderator of the pump pointing caudally (away from the incision site). The wound was closed using Autoclips (Kent Scientific, Torrington, CT) and sterilised with Vetasept. The animal was then liberated from the face mask, an analgesic (0.05 mg/kg buprenorphine/Vetergesic, Alstoe, UK) was administered subcutaneously and the mouse was placed into a preheated recovery cage with soft food readily available.

Blood Pressure Measurements

Blood pressure measurements were carried using the Letica LE 5002 Non-Invasive Blood Pressure System (Panlab, Cornellá, Spain). The system operates on the same basis as clinically used sphygmomanometers. By inflation of the cuff placed around the proximal part of the tail the arterial blood flow is occluded and subsequently during controlled deflation a sensor measures both systolic and diastolic BP.

Prior to the start of the experimental protocol mice were trained to tail cuff plethysmography in order to decrease the load of stress related to taking the measurements and to improve reproducibility. During the experimental period animals were subjected to plethysmography after every imaging session. Mice were placed into a restraining chamber while recovering from anaesthesia. The plethysmograph cuff which has an inbuilt sensor was positioned at the proximal end of the tail. To maintain body temperature during the measurements mice were kept in a preheated recovery box (MediHEAT, Vet-Tech solutions, Congleton, UK). After 2 preliminary measurements, at least three recordings of systolic, diastolic and mean arterial pressure per animal were recorded. The time between measurements was >10 seconds - which was the time required for the system to deflate the cuff.

Sacrifice and Tissue Collection

After the final imaging session animals were kept under anaesthesia and transferred to a face mask delivering 3% isoflurane in oxygen (flow 1/L/min). Through a midline incision of the abdomen and chest the ribcage and (after cutting the sternum) the heart was visualised. Animals were then euthanized by exsanguination via cardiac puncture. Death was confirmed by cessation of circulation. The heart was then removed and transferred into 10% neutral buffered formalin (Cellstor, CellPath, Newton, UK) for fixation for at least 20 hours.

Histological Analysis

After fixation hearts were processed in a tissue processor by immersing in graded ethanol washes (70%,95%,100%), Xylene and finally Paraffin wax. The wax infiltrated hearts were then placed in a mould containing molten paraffin which was allowed to cool. Samples were embedded in paraffin in a short axis orientation so that the sections cut would all show the ventricle lumen. For sectioning, heart tissue blocks were chilled on ice and then cut on microtome. Prior to histological staining, sections were deparaffinized and rehydrated at room temperature according to the following protocol. For deparaffinization, samples were immersed twice in xylene for 5 minutes. In the next step, slides were rehydrated by sequentially immersing in graded ethanol washes (100%, 95%, 85%,70 and 50%) for 3 minutes each. Subsequently, samples were washed in 0.85% NaCl for 5 minutes and immersed in phosphate buffered saline for 5 minutes. Slides were stained in a 0.1% sirius red solution in saturated picric acid (Picrosirius red, Sigma, Dorset, UK) in the dark for 90 minutes. Slides were then briefly washed three times in acidified water by quickly dipping 3 times and dehydrated by immersing in graded ethanol washes (50%, 70%, 85%, 95% 100%), cleared in xylene for 5 minutes and mounted with permanent mounting media (Pertex, CellPath,Newtown, UK). All picrosirius red stained slides images were acquired on the AxioScan Z1 (Carl Zeiiss, Oberkochen, Germany) using an in-house developed protocol for bright field mouse myocardium slides. The .CZI files were converted into tagged image file format (.TIFF) to facilitate image analysis using Image-Pro Premiere 9.1 (MediaCybernetics, Rockville, MD, USA). In the first step, the pixel area occupied by fibrosis was calculated using the counting tool after manual protocol adjustment. A pixel intensity threshold of 204-245 on the Mono scale was applied. The highlighted area was inspected for any contamination by erythrocytes or endo/epicardium and if present these objects were removed before the final output was recorded. In a similar fashion, the total area of the analysed tissue was calculated by considering the area within the threshold of 150-245 on the mono scale with the exceptions outlined above made if necessary. Finally, the area of fibrosis was expressed as a percentage of the slide total myocardium area.
